# OSBPL2 inhibition leads to apoptosis of cochlea hair cells in age-related hearing loss by inhibiting the AKT/FOXG1 signaling pathway

**DOI:** 10.18632/aging.206138

**Published:** 2024-10-30

**Authors:** Meina Li-Yang, Chao Ma, Xiaoye Wang, Jianqiang You

**Affiliations:** 1 Department of Otolaryngology, The First People’s Hospital of Changzhou, Jiangsu 213003, China; 2 Department of Cardiothoracic Surgery, The First People’s Hospital of Changzhou, Jiangsu 213003, China

**Keywords:** age-related hearing loss, OSBPL2, FOXG1, AKT, cochlea hair cells

## Abstract

Age-related hearing loss (AHL) is a prevalent and multifaceted condition that significantly impacts a substantial portion of the aging population. Oxysterol Binding Protein-like 2 (OSBPL2) has been identified as a causal gene for hearing loss. However, its role in AHL is still unclear. In this study, we investigated the effect of OSBPL2 on the survival of cochlea hair cells. To simulate AHL *in vitro*, hair cell-like inner ear cells (HEI-OC1) were exposed to H_2_O_2_ treatment. OSBPL2 expression was significantly increased in HEI-OC1 cells after H_2_O_2_ treatment. OSBPL2 knockdown augmented cell death and apoptosis in H_2_O_2_-induced HEI-OC1 cells. Besides, H_2_O_2_ treatment also led to the inactivation of the AKT and FOXG1 signaling pathways in HEI-OC1 cells. Mechanistically, OSBPL2 silencing reinforced the inactivation of the FOXG1 signaling pathway in H_2_O_2_-treated HEI-OC1 cells by inhibiting the AKT signaling pathway. Under H_2_O_2_ treatment, AKT inhibition by MK2206 augmented the apoptosis of HEI-OC1 cells; on the contrary, AKT activation by SC79 treatment partially rescued the apoptosis of OSBPL2-knockdown HEI-OC1 cells. In addition, FOXG1 silencing significantly reversed the effects of AKT activation on OSBPL2-knockdown HEI-OC1 cells. Moreover, OSBPL2 expression and the activation status of the AKT/FOXG1 signaling pathway were confirmed in the cochleae of young and old C57BL/6 mice. In conclusion, our study provides evidence that OSBPL2 inhibition sensitizes HEI-OC1 cells to H_2_O_2_-induced apoptosis via inactivation of the AKT/FOXG1 signaling pathway, suggesting that OSBPL2 acts as an important regulator in AHL.

## INTRODUCTION

Age-related hearing loss (AHL), also known as presbycusis, is the most frequently occurring sensory disorder affecting the aging population [1, 2]. AHL is characterized by a progressive decline in auditory function with aging, often manifesting as difficulty in hearing high-frequency sounds, speech comprehension, and increased sensitivity to background noise [3, 4]. This age-associated sensory impairment not only affects interpersonal communication but also has broader implications, contributing to social isolation, cognitive decline, and diminished overall well-being [5, 6]. Globally, the incidence of AHL is increasing due to the aging population, which has become a major burden on families and society [7].

Generally, AHL is associated with an age-dependent loss of sensory hair cells, spiral ganglion neurons, and stria vascularis cells in the inner ear [8]. Sensory hair cells, highly specialized sensory receptors located in the inner ear cochlea, are responsible for the mechano-transduction of sound waves into electrical signals [9]. Unfortunately, auditory HCs are naturally differentiated only during the embryonic development course in mammals and are unable to regenerate once damaged [10]. As a result, loss or degeneration of cochlea HCs may result in permanent and irreversible hearing loss [11]. In addition, apoptosis of cochlea HCs has been proposed to be the classic theory behind the AHL development [7, 12]. Thus, it is highly important to explore the intricate mechanism governing survival and apoptosis of cochlea HCs in ALH.

Oxysterol-binding protein like 2 (OSBPL2), a member of the oxysterol-binding protein (OSBP) family [13], plays a critical role in cellular lipid homeostasis and intracellular lipid transport. OSBPL2 is expressed in the developing mouse cochlea [14], suggesting its potential involvement in the intricate process of hair cell development. In addition, OSBPL2 has been identified as a novel causal gene for autosomal dominant non-syndromic hearing loss (ADNSHL) [15]. Furthermore, previous studies have shown that OSBPL2 knockout leads to severe hearing loss in mice, suggesting its crucial role in maintaining the auditory function of the inner ear [14, 16]. However, the role of OSBPL2 in cochlea HC apoptosis in ALH and the underlying molecular mechanisms remain largely unexplored.

In this work, we aimed to investigate the specific effects of OSBPL2 on the survival and apoptosis of H_2_O_2_-induced HEI-OC1 cells. The results indicated that OSBPL2 deficiency led to hearing loss in mice and caused HEI-OC1 cell apoptosis by inhibiting the AKT/FOXG1 signaling pathway. These findings provide a further understanding of the molecular function of OSBPL2 in the apoptosis of cochlea HCs.

## MATERIALS AND METHODS

### Cell culture and treatment

House Ear Institute-Organ of Corti 1 (HEI-OC1) cells were purchased from BioFeng (Shanghai, China). As previously described [17], HEI-OC1 cells were cultured under non-permissive (39° C; 5% CO_2_) conditions in high-glucose DMEM (10% FBS) without antibiotics.

HEI-OC1 cells were exposed to H_2_O_2_ at different concentrations (50, 100, 200, 400, 800, or 1000 μM) for 1 h [18]. Then, treated HEI-OC1 cells were washed with PBS and then cultured for the indicated time, and cell viability assays were performed under these experimental conditions.

### Cell transfection

Short hairpin RNA (shRNA; CAACAAGATGAAGAGCACCAA) targeting OSBPL2 (sh-OSBPL2; GCAGCCTTGGAATCCTCAAAT), shRNA targeting FOXG1 (sh-FOXG1; GTCTTCTTCCAACCCTTTAAT), negative control (shNC), FOXG1 pcDNA3.1 plasmid (FOXG1-OE), and empty pcDNA3.1 vector (Vector) were obtained from GenePharma (Shanghai, China). HEI-OC1 cells were transfected with the above plasmids via Lipofectamine® 3000 (Invitrogen, USA) at 37° C [19].

### CCK-8

CCK-8 assay was applied to assess HEI-OC1 cell viability [20]. Briefly, HEI-OC1 cells were cultivated in 96-well plates (5×10^3^ cells/well). After incubation for the indicated time, 10 μl CCK-8 was added to each well. Then, cell viability was determined via a microplate reader at 490 nm.

### TUNEL

HEI-OC1 cell apoptosis was assessed by Bright Green Apoptosis Detection kit (Vazyme, Nanjing, China) 24 h after H_2_O_2_ treatment [21]. In brief, cells were fixed in 4% paraformaldehyde, permeabilized with 0.1% Triton X-100, and then treated with TUNEL reaction mixture. TUNEL-positive cells were calculated under a fluorescence microscope.

### Western blotting

Total proteins were obtained from HEI-OC1 cells and cochleae. After concentration determination, total protein was separated by SDS-PAGE and electroblotted to PVDF membranes. Following blocking with 5% skim milk for 1 h, the membranes were probed with primary antibodies against OSBPL2 (ab235298; Abcam, UK), Bax (ab32503; Abcam), Bcl-2 (ab182858; Abcam), p-AKT (ab38449; Abcam), AKT (ab8805; Abcam), p-PI3K (ab278545; Abcam), PI3K (ab154598; Abcam), PDK1 (ab202468; Abcam), FOXG1 (ab196868; Abcam), WNT3A (ab219412; Abcam), FOXO1 (#2880; Cell Signaling Technology, USA), and GAPDH (ab9485; Abcam) overnight, and then probed with HRP-conjugated secondary antibody (ab6721; Abcam). Finally, the immunoreactive bands were visualized through the ECL plus Kit (Beyotime, Jiangsu, China) [22].

### Animal study

C57BL/6 mice were purchased from the Animal Model Institute of Nanjing (Nanjing, China). Mice were divided into Young (4 weeks old) and Old (12 months old) groups [23]. The mice were euthanized by cervical dislocation under 5% isoflurane anesthesia. Then, cochleae were isolated from mice for further studies.

### Auditory brainstem responses (ABR) testing

ABR measurements were performed as previously described [11]. In brief, mice were anesthetized with 0.007 g/ml pentobarbital sodium and were placed in a soundproof room. Needle electrodes were introduced just under the skin, with the active electrode placed between the ears above the vertex of the skull, the ground electrode between the eyes, and the reference electrode underneath the left ear. Mice were presented with click stimuli generated using a Tucker Davis Technologies (TDT) workstation running SigGen32 software (TDT). Auditory thresholds were determined by decreasing the sound intensities from 90 to 10 dB until the waveforms lost their reproducible morphology.

### Statistical analysis

Every experiment was repeated three times. All data were represented as mean±SD. Comparisons were severally assessed by Student’s t-test or one-way ANOVA. P< 0.05 was deemed significant.

### Availability of data and materials

The datasets used and/or analyzed during the current study are available from the corresponding author upon reasonable request.

## RESULTS

### OSBPL2 is associated with H_2_O_2_-induced cytotoxic effects on HEI-OC1 cells

**Figure 1:**
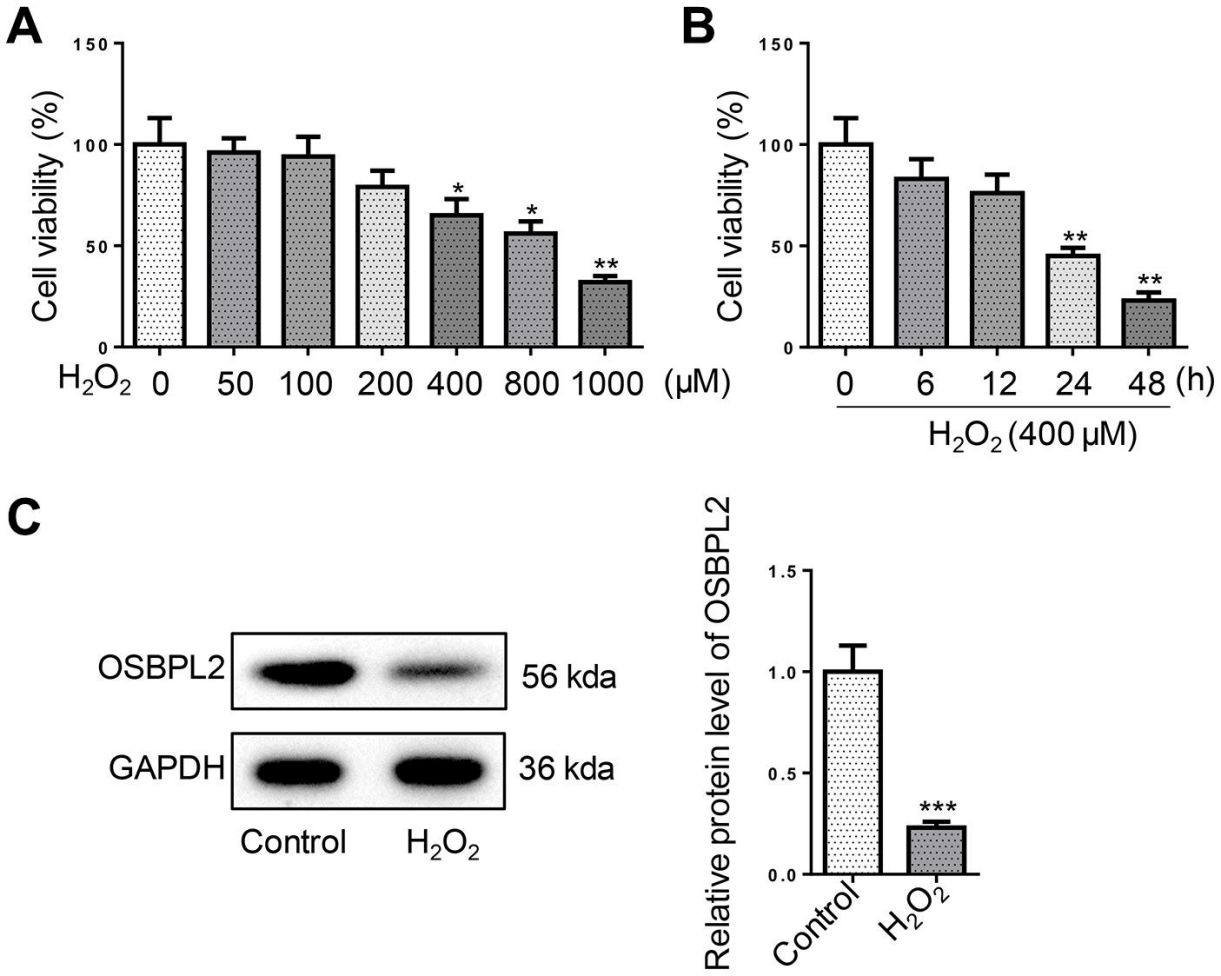
**OSBPL2 is associated with H_2_O_2_-induced cytotoxic effects on HEI-OC1 cells.** (**A**) HEI-OC1 cells were exposed to H_2_O_2_ for 1 h at designated concentrations (50, 100, 200, 400, 800, and 1000 μM). The viability of HEI-OC1 cells was measured by CCK-8 assay. (**B**) The viability of HEI-OC1 cells was measured by CCK-8 assay at the indicated time after H_2_O_2_ (400 μM) treatment. (**C**) OSBPL2 protein expression levels in HEI-OC1 cells from Control and H_2_O_2_ groups. All *in vitro* experiments were performed in triplicate (n = 3). *P<0.05; **P<0.01.

To simulate AHL *in vitro*, hair cell-like inner ear cells (HEI-OC1 cells) were exposed to H_2_O_2_ (50–1000 μM) for 1 h. As evidenced by CCK-8 assay, H_2_O_2_ treatment decreased cell viability of HEI-OC1 cells in a concentration-dependent manner (Figure 1A). In addition, 400 μM H_2_O_2_ treatment decreased cell viability of HEI-OC1 cells in a time-dependent manner (Figure 1B). Since HEI-OC1 cell viability was markedly reduced to about 50 % at 24 h after treatment with 400 μM H_2_O_2_, 400 μM was applied as the concentration of H_2_O_2_ treatment for subsequent functional assays. Western blotting results revealed that OSBPL2 expression was significantly decreased in HEI-OC1 cells after H_2_O_2_ treatment (Figure 1C). These data suggested that OSBPL2 might be associated with H_2_O_2_-induced HEI-OC1 cell damage.

### OSBPL2 absence induces apoptosis in H_2_O_2_-treated HEI-OC1 cells

**Figure 2:**
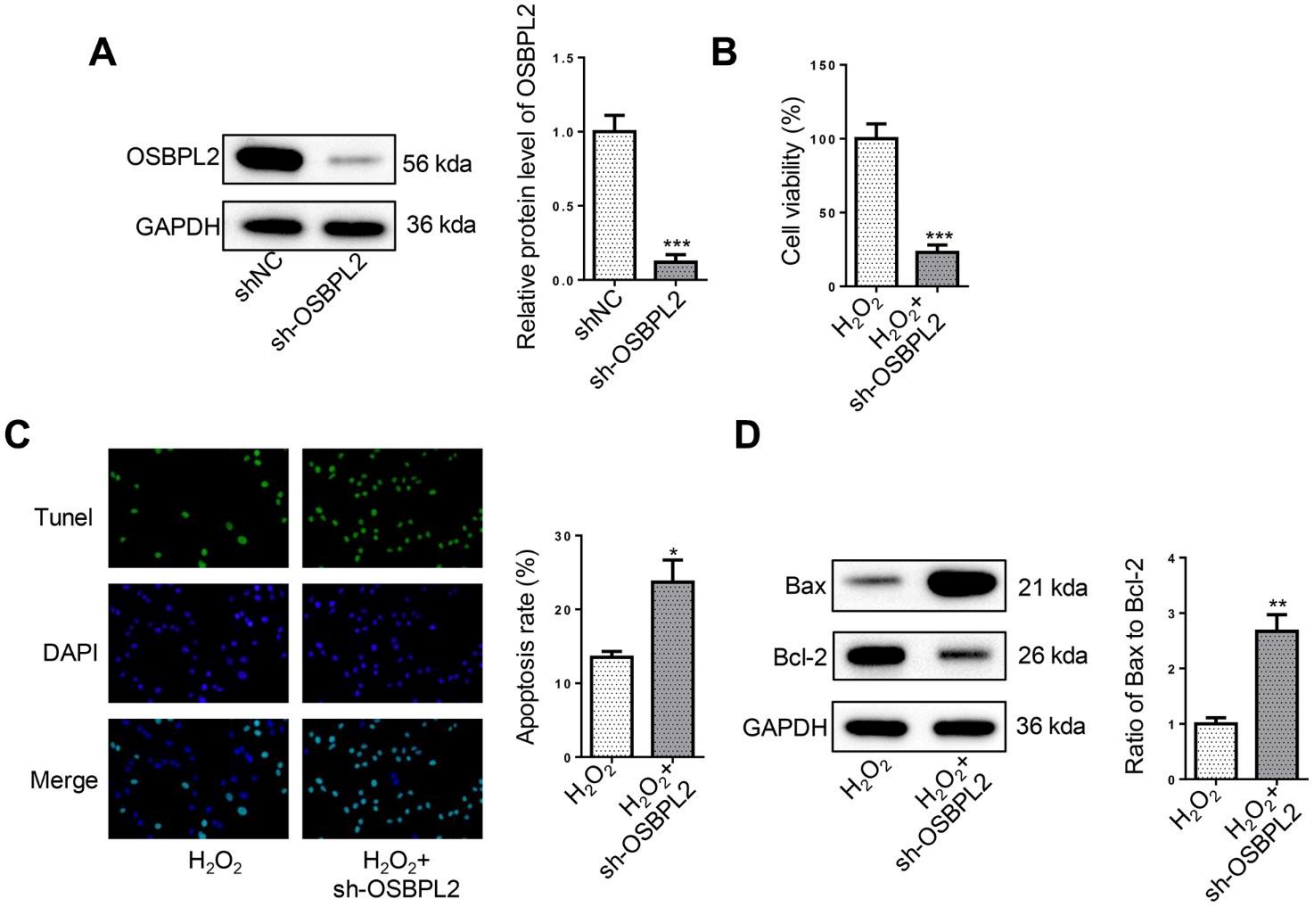
**OSBPL2 absence induces apoptosis in H_2_O_2_-treated HEI-OC1 cells.** (**A**) OSBPL2 protein levels in HEI-OC1 cells transfected with shNC and sh-OSBPL2. (**B**) CCK-8 assay for the viability of HEI-OC1 cells from H_2_O_2_ and H_2_O_2_+sh-OSBPL2 groups. (**C**) TUNEL assay for the apoptosis of HEI-OC1 cells from each group. (**D**) Bax and Bcl-2 protein levels in HEI-OC1 cells from each group. All *in vitro* experiments were performed in triplicate (n = 3). * P<0.05; ** P<0.01.

To investigate the effects of OSBPL2 disruption on the survival and apoptosis of H_2_O_2_-treated HEI-OC1 cells, OSBPL2 was first knocked down in HEI-OC1 cells and the efficiency was confirmed by western blotting (Figure 2A). CCK-8 results showed that OSBPL2 knockdown significantly inhibited the survival of H_2_O_2_-induced HEI-OC1 cells (Figure 2B). As illustrated by TUNEL results, OSBPL2 inhibition remarkably increased the apoptotic rate of HEI-OC1 cells in the presence of H_2_O_2_ (Figure 2C). Western blotting results further confirmed that OSBPL2 knockdown led to a significant increase in Bax/Bcl-2 ratio in H_2_O_2_-challenged HEI-OC1 cells (Figure 2D). Therefore, OSBPL2 knockdown promoted HEI-OC1 cell apoptosis.

### OSBPL2 deletion inactivates AKT signaling pathway

**Figure 3:**
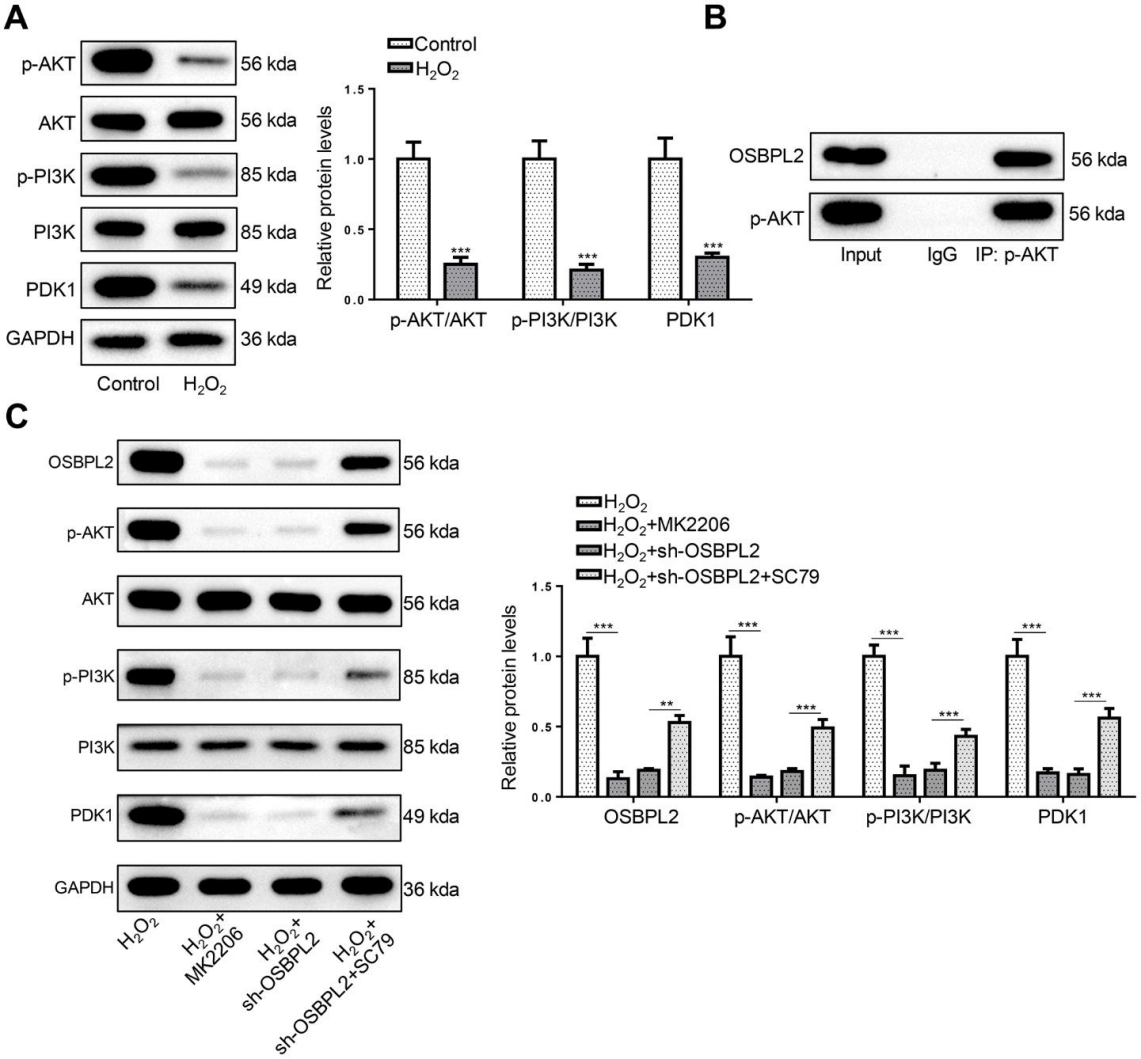
**OSBPL2 deletion inactivates AKT signaling pathway.** (**A**) The levels of AKT-related proteins (p-AKT, AKT, p-PI3K, PI3K, and PDK1) in HEI-OC1 cells from Control and H_2_O_2_ groups. (**B**) Co-IP assay for OSBPL2 and p-AKT in HEI-OC1 cells. (**C**) The levels of OSBPL2 and AKT-related proteins in HEI-OC1 cells from H_2_O_2_, H_2_O_2_+MK2206, H_2_O_2_+sh-OSBPL2, and H_2_O_2_+sh-OSBPL2+SC79 groups. All *in vitro* experiments were performed in triplicate (n = 3). * P<0.05; ** P<0.01.

The inhibition of the PI3K/AKT signaling pathway is closely related to the development of AHL [24]. In addition, the PI3K/AKT signaling pathway development and survival of cochlea HCs [10]. Therefore, the expression of OSBPL2 and related proteins in the AKT signaling pathway (p-AKT, AKT, p-PI3K, PI3K, and PDK1) was detected by western blotting. Western blotting results showed that p-AKT, p-PI3K, and PDK1 protein levels were decreased in H_2_O_2_-treated HEI-OC1 cells, compared with normal HEI-OC1 cells (Figure 3A). Next, the interaction between OSBPL2 and p-AKT was assessed by co-IP assay. As shown in Figure 3B, there was an interaction between OSBPL2 and p-AKT in HEI-OC1 cells. To verify the interaction between OSBPL2 and p-AKT, an AKT inhibitor (MK2206) and an AKT activator (SC79) were applied to treat HEI-OC1 cells. H_2_O_2_, H_2_O_2_+MK2206, H_2_O_2_+sh-OSBPL2, and H_2_O_2_+sh-OSBPL2+SC79 groups. Western blotting results showed that MK2206 significantly inhibited the expression levels of OSBPL2 and AKT-related proteins in HEI-OC1 cells; SC79 upregulated OSBPL2 expression in OSBPL2-silenced HEI-OC1 cells and partially reversed the inhibition of the AKT signaling pathway mediated by OSBPL2 deletion (Figure 3C). These results indicated that OSBPL2 knockdown led to AKT inactivation *in vitro*.

### OSBPL2 inhibition causes HEI-OC1 cell apoptosis by inhibiting AKT activation

**Figure 4:**
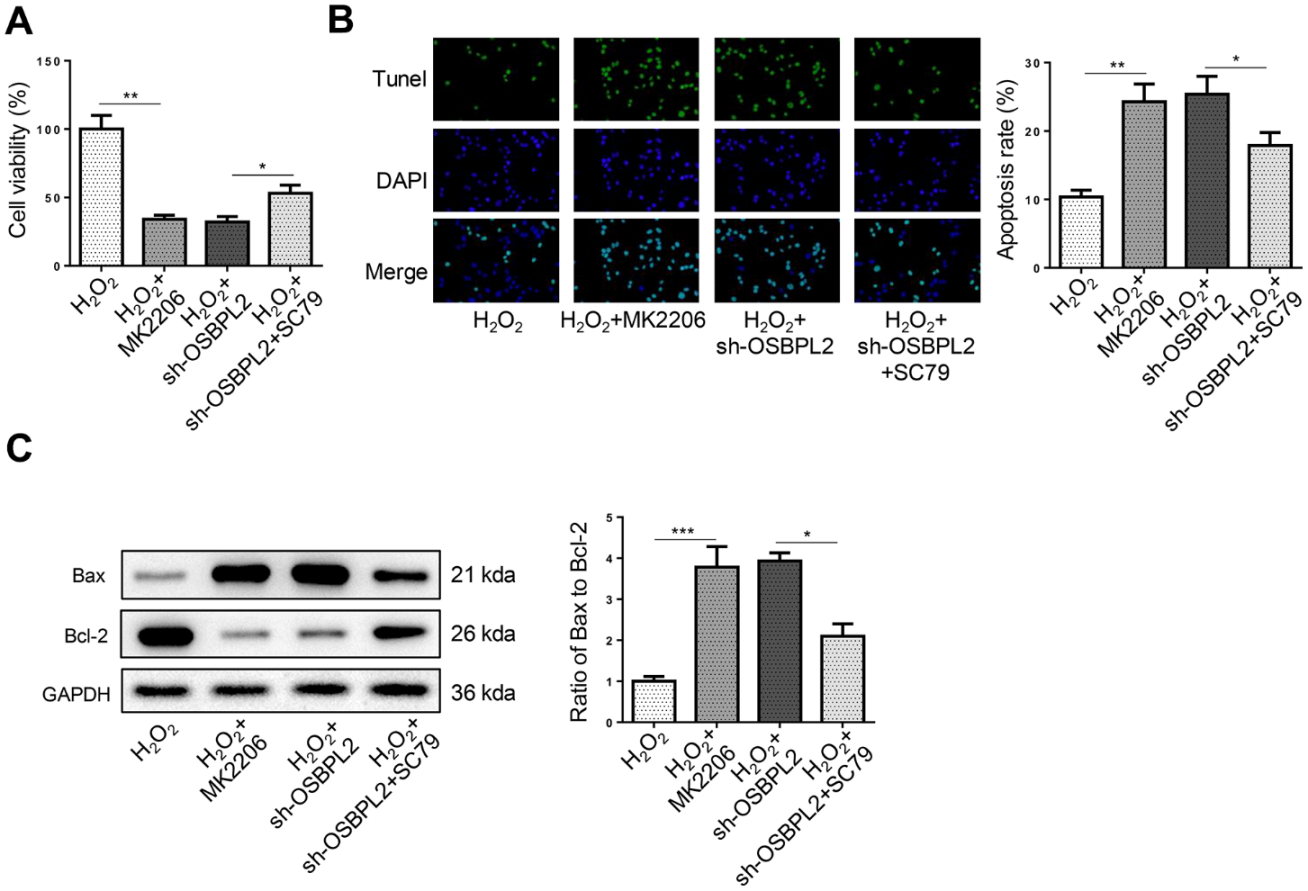
**OSBPL2 inhibition causes HEI-OC1 cell apoptosis by inhibiting AKT activation.** (**A**) The cell viability of HEI-OC1 cells from H_2_O_2_, H_2_O_2_+MK2206, H_2_O_2_+sh-OSBPL2, and H_2_O_2_+sh-OSBPL2+SC79 groups. (**B**) The cell apoptosis of HEI-OC1 cells from each group. (**C**) Bax and Bcl-2 protein levels in HEI-OC1 cells from each group. All *in vitro* experiments were performed in triplicate (n = 3). * P<0.05; ** P<0.01.

Next, the effects of the AKT signaling on HEI-OC1 cell apoptosis were further evaluated. CCK-8 and TUNEL results showed that MK2206 reduced the viability of HEI-OC1 cells and promoted HEI-OC1 cell apoptosis, whereas SC79 treatment abrogated the effects of OSBPL2 silencing on HEI-OC1 cell viability and apoptosis (Figure 4A, 4B), which was further confirmed by the changes in Bax/Bcl-2 rate in HEI-OC1 cells (Figure 4C). These results indicated that AKT activation reversed OSBPL2 knockdown-mediated HEI-OC1 cell apoptosis.

### OSBPL2 deficiency inhibits the FOXG1 signaling pathway in H_2_O_2_-treated HEI-OC1 cells via AKT inactivation

**Figure 5:**
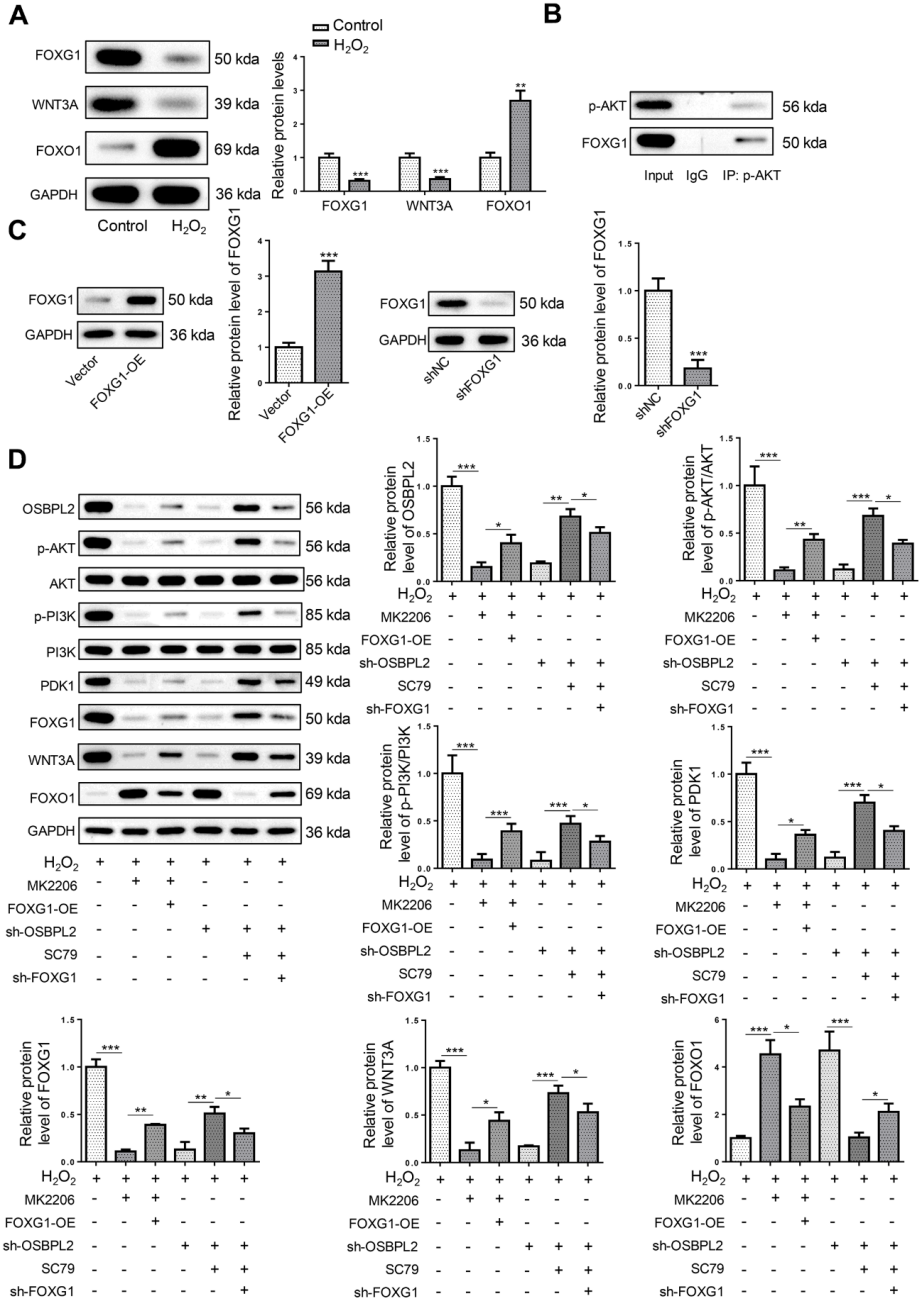
**OSBPL2 deficiency inhibits the FOXG1 signaling pathway in H_2_O_2_-treated HEI-OC1 cells via AKT inactivation.** (**A**) The levels of FOXG1-related proteins (FOXG1, WNT3A, and FOXO1) in HEI-OC1 cells from Control and H_2_O_2_ groups. (**B**) Co-IP assay for p-AKT and FOXG1 in HEI-OC1 cells. (**C**) FOXG1 protein level in HEI-OC1 cells transfected with Vector, FOXG1-OE, shNC or sh-FOXG1. (**D**) The levels of OSBPL2 and AKT/FOXG1-related proteins in H_2_O_2_, H_2_O_2_+MK2206, H_2_O_2_+MK2206+FOXG1-OE, H_2_O_2_+sh-OSBPL2, H_2_O_2_+sh-OSBPL2+SC79, and H_2_O_2_+sh-OSBPL2+SC79+sh-FOXG1 groups. All *in vitro* experiments were performed in triplicate (n = 3). * P<0.05; ** P<0.01.

Besides, FOXG1 plays a key role in hair cell development and survival [25, 26]. Therefore, related proteins of the FOXG1 signaling pathway (FOXG1, WNT3A, and FOXO1) were detected. It was shown that OSBPL2 silencing suppressed FOXG1 and WNT3A protein levels and increased FOXO1 protein level in H_2_O_2_-challenged HEI-OC1 cells (Figure 5A). Interestingly, AKT has been reported as an upstream regulator of FOXG1 signaling pathway [27]. Therefore, OSBPL2 might regulate the FOXG1 signaling pathway via the AKT signaling pathway. The results of Co-IP assay revealed an interaction between p-AKT and FOXG1 (Figure 5B), indicating that OSBPL2 regulated FOXG1 expression via AKT inactivation.

To further verify the relationship between OSBPL2, AKT signaling pathway, and FOXG1, HEI-OC1 cells were assigned to H_2_O_2_, H_2_O_2_+MK2206, H_2_O_2_+MK2206+FOXG1-OE, H_2_O_2_+sh-OSBPL2, H_2_O_2_+sh-OSBPL2+SC79, and H_2_O_2_+sh-OSBPL2+SC79+sh-FOXG1 groups. First, FOXG1 overexpression and knockdown efficiencies were verified by western blotting (Figure 5C). Western blotting results showed that FOXG1 overexpression reversed MK2206-induced inhibition of AKT and FOXG1 signaling pathways in H_2_O_2_-induced HEI-OC1 cells; moreover, FOXG1 silencing partially abated the effects of SC79 on OSBPL2 knockdown-mediated inactivation of AKT and FOXG1 signaling pathways in H_2_O_2_-challenged HEI-OC1 cells (Figure 5D). These results suggested that OSBPL2 activated the FOXG1 signaling pathway by activating the AKT signaling pathway in HEI-OC1 cells.

### OSBPL2 deletion promotes H_2_O_2_-induced HEI-OC1 cell apoptosis by downregulating FOXG1 expression

**Figure 6:**
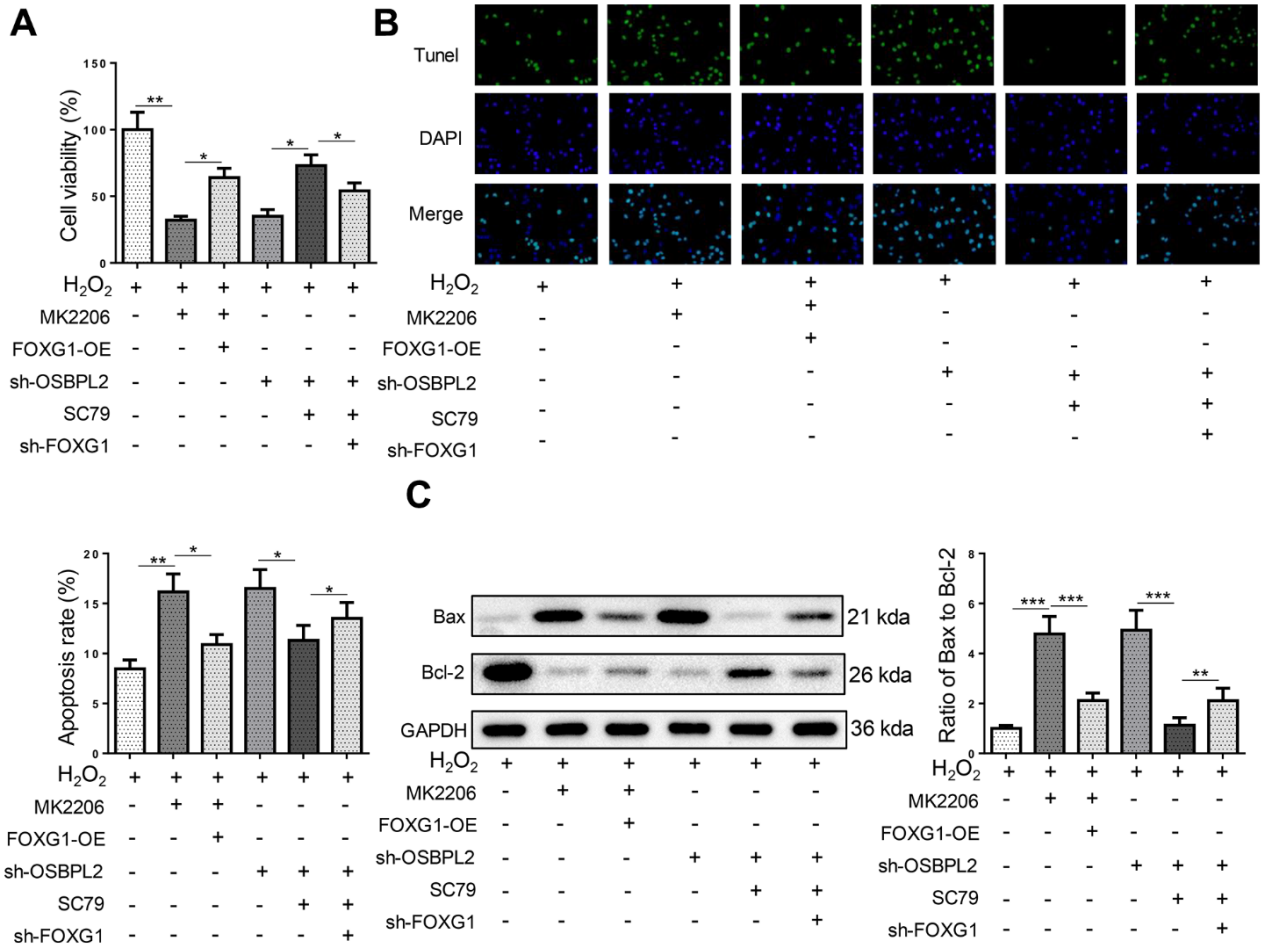
**OSBPL2 deletion promotes H_2_O_2_-induced HEI-OC1 cell apoptosis by downregulating FOXG1 expression.** (**A**) The cell viability of HEI-OC1 cells from H_2_O_2_, H_2_O_2_+MK2206, H_2_O_2_+MK2206+FOXG1-OE, H_2_O_2_+sh-OSBPL2, H_2_O_2_+sh-OSBPL2+SC79, and H_2_O_2_+sh-OSBPL2+SC79+sh-FOXG1 groups. (**B**) The cell apoptosis of HEI-OC1 cells from each group. (**C**) Bax and Bcl-2 protein levels in HEI-OC1 cells from each group. All *in vitro* experiments were performed in triplicate (n = 3). * P<0.05; ** P<0.01.

Next, the impact of FOXG1 on H_2_O_2_-induced HEI-OC1 cells was assessed. It was shown that FOXG1 silencing significantly reversed the mitigatory effects of SC79 on HEI-OC1 cell apoptosis caused by OSBPL2 knockdown (Figure 6A–6C). Therefore, OSBPL2 inhibition promoted HEI-OC1 cell apoptosis by downregulating FOXG1 expression via AKT deactivation.

### OSBPL2 downregulation and inactivation of AKT/FOXG1 signaling pathways in the cochleae of aged C57BL/6 mice

**Figure 7:**
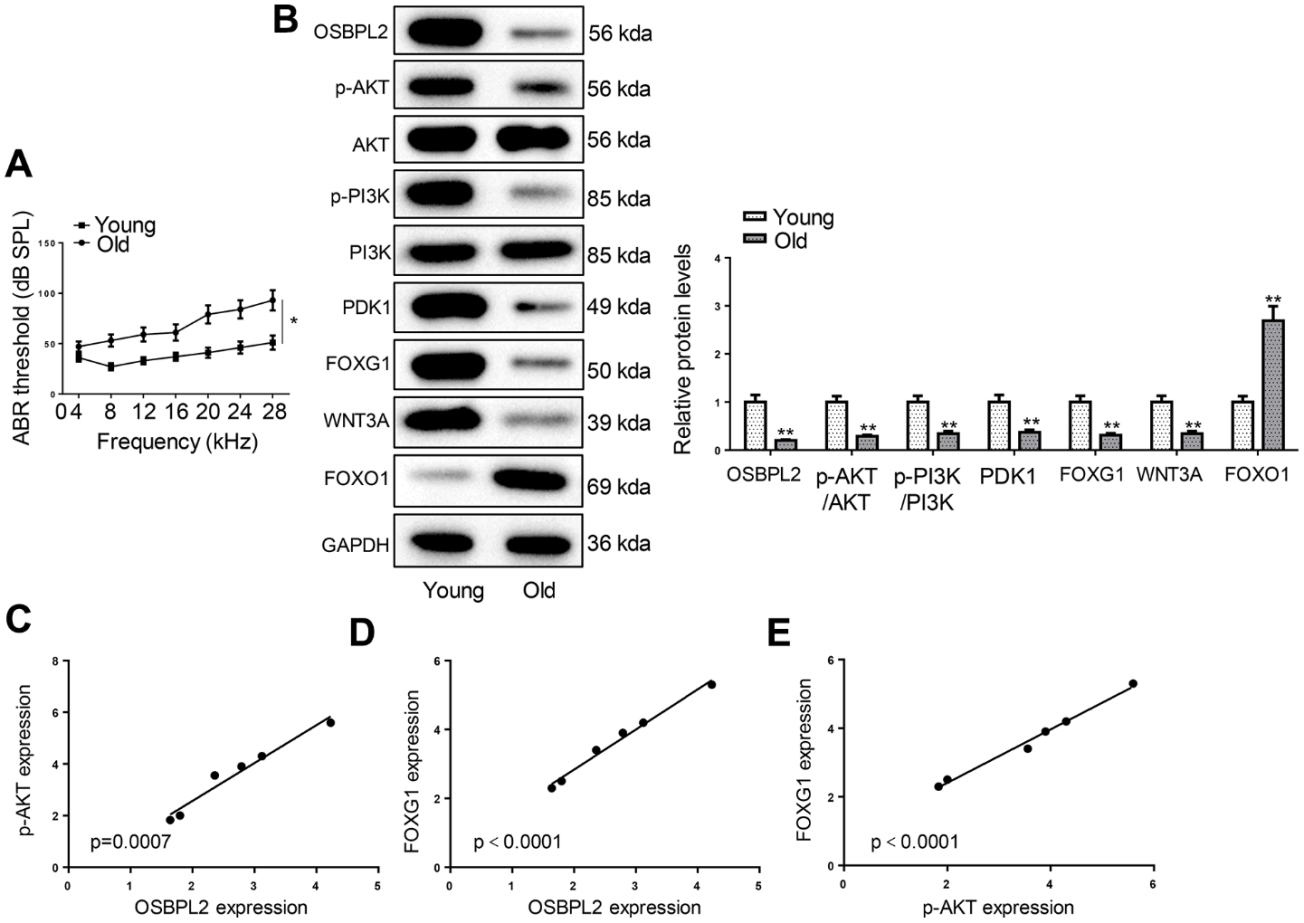
**OSBPL2 downregulation and inactivation of AKT/FOXG1 signaling pathways in the cochleae of aged C57BL/6 mice.** (**A**) ABR thresholds of C57BL/6 mice from Young (n=6) and Old (n=6) groups in response to tone pips of 8, 12, 16, 20, 24, 28, and 32 kHz. (**B**) Western blotting for expression levels of OSBPL2 and proteins related to AKT and FOXG1 signaling pathways in the cochleae of young and old C57BL/6 mice. (**C**, **D**) Association between OSBPL2 and p-AKT or FOXG1 expression in the cochleae of aged C57BL/6 mice. (**E**) Association between p-AKT and FOXG1 expression in the cochleae of aged C57BL/6 mice. * P<0.05; ** P<0.01.

C57BL/6 mice are recognized for their propensity for early-onset hearing loss, making them commonly employed as *in vivo* models for AHL [23]. To examine the hearing function of C57BL/6 mice, we performed ABR tests on young (4 weeks old) and old (12 months old) C57BL/6 mice. As shown in Figure 7A, the ABR thresholds of old mice were significantly higher than those of young mice; In addition, the hearing loss also appeared severe at a higher frequency range, indicating a decline of auditory function in aged mice. Then, the expression levels of OSBPL2, AKT-related proteins, and FOXG1-related proteins were detected in the cochleae of young and old C57BL/6 mice. Consistent with the results obtained from *in vitro* assays, aged C57BL/6 mice exhibited significantly lower OSBPL2 expression and inactivation of the AKT and FOXG1 signaling pathways (Figure 7B). OSBPL2 expression was positively correlative to p-AKT and FOXG1 expression (Figure 7C, 7D). In addition, p-AKT expression was also positively correlated to FOXG1 expression (Figure 7E). These results further supported our *in vitro* findings that OSBPL2 inhibition contributed to AHL via the AKT/FOXG1 signaling pathway.

## DISCUSSION

Due to the increasing incidence in recent years, AHL has become a major issue for individuals and society [28]. Apoptosis is known as a programmed form of cell death [29]. Accelerated apoptosis of cochlea hair cells is considered a key factor leading to AHL [30]. Therefore, HEI-OC1 cells were exposed to H_2_O_2_ treatment to imitate AHL *in vitro* in this study. HEI-OC1 cell apoptosis was investigated to uncover the underlying mechanism of AHL.

OSBPL2 has been identified as a novel candidate gene for progressive non-syndromic hearing loss, autosomal dominant hearing loss, and ADNSHL [15, 16, 31]. In addition, OSBPL2 deletion may also lead to dysfunction of auditory cells by promoting excessive cholesterol biosynthesis and reactive oxygen species (ROS) production, indicating OSBPL2 deficiency may be implicated in the pathogenesis of sensorineural hearing loss [13]. However, the role of OSBPL2 in AHL has not been previously reported. Our results revealed a decline of OSBPL2 expression in H_2_O_2_-treated HEI-OC1 cells, compared with normal HEI-OC1 cells. Another study demonstrated that OSBPL2-knockout pigs exhibited increased apoptosis of cochlea hair cells [32], suggesting a crucial regulatory role of OSBPL2 in cochlea hair cell apoptosis. As expected, OSBPL2 knockdown in HEI-OC1 cells potentiated the decrease of cell survival and the increase of cell apoptosis caused by H_2_O_2_ treatment, which was also evidenced by the reinforced Bax/Bcl-2 ratio in HEI-OC1 cells. To sum up, OSBPL2 plays a crucial role in the regulation of the apoptosis of H_2_O_2_-induced HEI-OC1 cells.

Recent studies suggest that the inhibition of the PI3K/AKT signaling pathway is associated with hearing loss following a variety of insults and stimuli, including AHL [24, 33, 34]. To unravel the molecular mechanisms underlying the role of OSBPL2 in the survival of H_2_O_2_-induced HEI-OC1 cells, the involvement of AKT and FOXG1 was investigated. In the present work, H_2_O_2_ induction led to inactivation of AKT and FOXG1 signaling pathways *in vitro*, which was augmented by OSBPL2 knockdown. Co-IP assay showed an interaction between OSBPL2 and p-AKT in HEI-OC1 cells. Under H_2_O_2_ treatment, AKT inhibition by MK2206 significantly reduced OSBPL2 expression in HEI-OC1 cells, while AKT activation by SC79 partly reversed the inhibitory effects on OSBPL2 expression and AKT activation mediated by OSBPL2 depletion. The PI3K/AKT signaling pathway has been reported to participate in the survival of cochlea HCs [25, 35, 36]. Consistent with the above findings, AKT inactivation increased the apoptotic rate of H_2_O_2_-induced HEI-OC1 cells; AKT activation partially counteracted the detrimental effects of OSBPL2 deficiency on HEI-OC1 cells exposed to H_2_O_2_ treatment. FOXG1 plays an important role in inner ear morphogenesis [37, 38]. Interestingly, AKT has been reported as a positive regulator of the FOXG1 signaling pathway [27]. Consistently, the interaction between p-AKT and FOXG1 was confirmed in HEI-OC1 cells. Additionally, FOXG1 overexpression reversed MK2206-induced inhibition of AKT/FOXG1 signaling pathways in H_2_O_2_-treated HEI-OC1 cells; moreover, FOXG1 silencing also abated SC79-induced activation of AKT/FOXG1 signaling pathways in OSBPL2-silenced HEI-OC1 cells. Taken together, OSBPL2 positively regulated the FOXG1 signaling pathway via AKT activation in H_2_O_2_-treatment HEI-OC1 cells. FOXG1 is deeply involved in cellular processes, including cell growth, development, apoptosis, and aging [39]. Furthermore, FOXGl has been reported to promote inner hair cell survival in AHL [40, 41]. Herein, FOXG1 silencing markedly counteracted the effects of SC79 on the promoting effects of OSBPL2 knockdown on H_2_O_2_-induced HEI-OC1 cell apoptosis. These results suggested that AKT/FOXG1 activation can counteract the detrimental effects of OSBPL2 deficiency and promote the survival of cochlea HCs in response to H_2_O_2_ treatment.

In conclusion, this study provides evidence for the involvement of OSBPL2 in the survival and apoptosis of cochlea HCs in AHL. Our findings suggested that OSBPL2 depletion potentiated H_2_O_2_-induced HEI-OC1 cell apoptosis by inhibiting the AKT/FOXG1 signaling pathway, implying therapeutic interventions targeting OSBPL2 and the AKT/FOXG1 pathway may be potential strategies for future treatment of AHL.

## References

[1] Li H, Lu M, Zhang H, Wang S, Wang F, Ma X, Liu J, Li X, Yang H, Shen H, Lv P. Downregulation of REST in the cochlea contributes to age-related hearing loss via the p53 apoptosis pathway. Cell Death Dis. 2022; 13:343. 10.1038/s41419-022-04774-0 35418568 PMC9007975

[2] Gates GA, Mills JH. Presbycusis. Lancet. 2005; 366:1111–20. 10.1016/S0140-6736(05)67423-5 16182900

[3] Zhao H, Wang X, Shi Y. The effect of hearing impairment and social participation on depressive symptoms in older adults: a cross-lagged analysis. Front Comput Neurosci. 2023; 17:1240587. 10.3389/fncom.2023.1240587 37614610 PMC10442535

[4] Yamasoba T, Someya S, Yamada C, Weindruch R, Prolla TA, Tanokura M. Role of mitochondrial dysfunction and mitochondrial DNA mutations in age-related hearing loss. Hear Res. 2007; 226:185–93. 10.1016/j.heares.2006.06.004 16870370

[5] Dawes P, Platt H, Horan M, Ollier W, Munro K, Pendleton N, Payton A. No association between apolipoprotein E or N-acetyltransferase 2 gene polymorphisms and age-related hearing loss. Laryngoscope. 2015; 125:E33–8. 10.1002/lary.24898 25155015 PMC4758402

[6] Badache AC, Mäki-Torkko E, Widen S, Fors S. Why Are Old-Age Disabilities Decreasing in Sweden and Denmark? Evidence on the Contribution of Cognition, Education, and Sensory Functions. J Gerontol B Psychol Sci Soc Sci. 2023; 78:483–95. 10.1093/geronb/gbac118 36112366 PMC9985323

[7] Sang L, Zheng T, Min L, Zhang X, Ma X, Entenman S, Su Y, Zheng Q. Otoprotective effects of ethosuximide in NOD/LtJ mice with age-related hearing loss. Int J Mol Med. 2017; 40:146–54. 10.3892/ijmm.2017.3004 28560432 PMC5466398

[8] Bao J, Ohlemiller KK. Age-related loss of spiral ganglion neurons. Hear Res. 2010; 264:93–7. 10.1016/j.heares.2009.10.009 19854255 PMC2868093

[9] Gálvez H, Abelló G, Giraldez F. Signaling and Transcription Factors during Inner Ear Development: The Generation of Hair Cells and Otic Neurons. Front Cell Dev Biol. 2017; 5:21. 10.3389/fcell.2017.00021 28393066 PMC5364141

[10] Kucharava K, Sekulic-Jablanovic M, Horvath L, Bodmer D, Petkovic V. Pasireotide protects mammalian cochlear hair cells from gentamicin ototoxicity by activating the PI3K-Akt pathway. Cell Death Dis. 2019; 10:110. 10.1038/s41419-019-1386-7 30728348 PMC6365508

[11] Zheng Z, Zeng S, Liu C, Li W, Zhao L, Cai C, Nie G, He Y. The DNA methylation inhibitor RG108 protects against noise-induced hearing loss. Cell Biol Toxicol. 2021; 37:751–71. 10.1007/s10565-021-09596-y 33723744 PMC8490244

[12] Guo L, Cao W, Niu Y, He S, Chai R, Yang J. Autophagy Regulates the Survival of Hair Cells and Spiral Ganglion Neurons in Cases of Noise, Ototoxic Drug, and Age-Induced Sensorineural Hearing Loss. Front Cell Neurosci. 2021; 15:760422. 10.3389/fncel.2021.760422 34720884 PMC8548757

[13] Wang H, Lin C, Yao J, Shi H, Zhang C, Wei Q, Lu Y, Chen Z, Xing G, Cao X. Deletion of OSBPL2 in auditory cells increases cholesterol biosynthesis and drives reactive oxygen species production by inhibiting AMPK activity. Cell Death Dis. 2019; 10:627. 10.1038/s41419-019-1858-9 31427568 PMC6700064

[14] Shi H, Wang H, Zhang C, Lu Y, Yao J, Chen Z, Xing G, Wei Q, Cao X. Mutations in OSBPL2 cause hearing loss associated with primary cilia defects via sonic hedgehog signaling. JCI Insight. 2022; 7:e149626. 10.1172/jci.insight.149626 35041619 PMC8876550

[15] Xing G, Yao J, Wu B, Liu T, Wei Q, Liu C, Lu Y, Chen Z, Zheng H, Yang X, Cao X. Identification of OSBPL2 as a novel candidate gene for progressive nonsyndromic hearing loss by whole-exome sequencing. Genet Med. 2015; 17:210–8. 10.1038/gim.2014.90 25077649

[16] Koh YI, Oh KS, Kim JA, Noh B, Choi HJ, Joo SY, Rim JH, Kim HY, Kim DY, Yu S, Kim DH, Lee SG, Jung J, et al. OSBPL2 mutations impair autophagy and lead to hearing loss, potentially remedied by rapamycin. Autophagy. 2022; 18:2593–614. 10.1080/15548627.2022.2040891 35253614 PMC9629061

[17] García-Mato Á, Cervantes B, Rodríguez-de la Rosa L, Varela-Nieto I. IGF-1 Controls Metabolic Homeostasis and Survival in HEI-OC1 Auditory Cells through AKT and mTOR Signaling. Antioxidants (Basel). 2023; 12:233. 10.3390/antiox12020233 36829792 PMC9952701

[18] Rivas-Chacón LD, Martínez-Rodríguez S, Madrid-García R, Yanes-Díaz J, Riestra-Ayora JI, Sanz-Fernández R, Sánchez-Rodríguez C. Role of Oxidative Stress in the Senescence Pattern of Auditory Cells in Age-Related Hearing Loss. Antioxidants (Basel). 2021; 10:1497. 10.3390/antiox10091497 34573129 PMC8464759

[19] Yu XZ, Yang BW, Ao MY, Wu YK, Ye H, Wang RY, Xi MR, Hou MM. CircNFIX stimulates the proliferation, invasion, and stemness properties of ovarian cancer cells by enhancing SH3RF3 mRNA stability via binding LIN28B. Kaohsiung J Med Sci. 2023; 39:234–43. 10.1002/kjm2.12632 36495291 PMC11895975

[20] You W, Zhang X, Ji M, Yu Y, Chen C, Xiong Y, Liu Y, Sun Y, Tan C, Zhang H, Li J, Chen W, Li R. MiR-152-5p as a microRNA passenger strand special functions in human gastric cancer cells. Int J Biol Sci. 2018; 14:644–53. 10.7150/ijbs.25272 29904279 PMC6001653

[21] Cao W, Zhang J, Wang G, Lu J, Wang T, Chen X. Reducing-Autophagy Derived Mitochondrial Dysfunction during Resveratrol Promotes Fibroblast-Like Synovial Cell Apoptosis. Anat Rec (Hoboken). 2018; 301:1179–88. 10.1002/ar.23798 29461680

[22] Pei J, Deng J, Ye Z, Wang J, Gou H, Liu W, Zhao M, Liao M, Yi L, Chen J. Absence of autophagy promotes apoptosis by modulating the ROS-dependent RLR signaling pathway in classical swine fever virus-infected cells. Autophagy. 2016; 12:1738–58. 10.1080/15548627.2016.1196318 27463126 PMC5079672

[23] Su Z, Xiong H, Liu Y, Pang J, Lin H, Zhang W, Zheng Y. Transcriptomic analysis highlights cochlear inflammation associated with age-related hearing loss in C57BL/6 mice using next generation sequencing. PeerJ. 2020; 8:e9737. 10.7717/peerj.9737 32879802 PMC7443093

[24] Chen X, Zhao X, Cai H, Sun H, Hu Y, Huang X, Kong W, Kong W. The role of sodium hydrosulfide in attenuating the aging process via PI3K/AKT and CaMKKβ/AMPK pathways. Redox Biol. 2017; 987–1003. 10.1016/j.redox.2017.04.031 28499253 PMC5429232

[25] He Z, Fang Q, Li H, Shao B, Zhang Y, Zhang Y, Han X, Guo R, Cheng C, Guo L, Shi L, Li A, Yu C, et al. The role of FOXG1 in the postnatal development and survival of mouse cochlear hair cells. Neuropharmacology. 2019; 144:43–57. 10.1016/j.neuropharm.2018.10.021 30336149

[26] Zhang S, Zhang Y, Dong Y, Guo L, Zhang Z, Shao B, Qi J, Zhou H, Zhu W, Yan X, Hong G, Zhang L, Zhang X, et al. Knockdown of Foxg1 in supporting cells increases the trans-differentiation of supporting cells into hair cells in the neonatal mouse cochlea. Cell Mol Life Sci. 2020; 77:1401–19. 10.1007/s00018-019-03291-2 31485717 PMC7113235

[27] Dastidar SG, Landrieu PM, D’Mello SR. FoxG1 promotes the survival of postmitotic neurons. J Neurosci. 2011; 31:402–13. 10.1523/JNEUROSCI.2897-10.2011 21228151 PMC4640444

[28] Gessa E, Giovanelli E, Spinella D, Verdelet G, Farnè A, Frau GN, Pavani F, Valzolgher C. Spontaneous head-movements improve sound localization in aging adults with hearing loss. Front Hum Neurosci. 2022; 16:1026056. 10.3389/fnhum.2022.1026056 36310849 PMC9609159

[29] Fuchs Y, Steller H. Live to die another way: modes of programmed cell death and the signals emanating from dying cells. Nat Rev Mol Cell Biol. 2015; 16:329–44. 10.1038/nrm3999 25991373 PMC4511109

[30] Li L, Xu K, Bai X, Wang Z, Tian X, Chen X. UCHL1 regulated by Sp1 ameliorates cochlear hair cell senescence and oxidative damage. Exp Ther Med. 2023; 25:94. 10.3892/etm.2023.11793 36761006 PMC9905655

[31] Thoenes M, Zimmermann U, Ebermann I, Ptok M, Lewis MA, Thiele H, Morlot S, Hess MM, Gal A, Eisenberger T, Bergmann C, Nürnberg G, Nürnberg P, et al. OSBPL2 encodes a protein of inner and outer hair cell stereocilia and is mutated in autosomal dominant hearing loss (DFNA67). Orphanet J Rare Dis. 2015; 10:15. 10.1186/s13023-015-0238-5 25759012 PMC4334766

[32] Yao J, Zeng H, Zhang M, Wei Q, Wang Y, Yang H, Lu Y, Li R, Xiong Q, Zhang L, Chen Z, Xing G, Cao X, Dai Y. OSBPL2-disrupted pigs recapitulate dual features of human hearing loss and hypercholesterolaemia. J Genet Genomics. 2019; 46:379–87. 10.1016/j.jgg.2019.06.006 31451425

[33] Chen J, Yuan H, Talaska AE, Hill K, Sha SH. Increased Sensitivity to Noise-Induced Hearing Loss by Blockade of Endogenous PI3K/Akt Signaling. J Assoc Res Otolaryngol. 2015; 16:347–56. 10.1007/s10162-015-0508-x 25790950 PMC4417093

[34] Xia W, Hu J, Ma J, Huang J, Jing T, Deng L, Zhang J, Jiang N, Ma D, Ma Z. Mutations in TOP2B cause autosomal-dominant hereditary hearing loss via inhibition of the PI3K-Akt signalling pathway. FEBS Lett. 2019; 593:2008–18. 10.1002/1873-3468.13482 31198993

[35] Aburto MR, Magariños M, Leon Y, Varela-Nieto I, Sanchez-Calderon H. AKT signaling mediates IGF-I survival actions on otic neural progenitors. PLoS One. 2012; 7:e30790. 10.1371/journal.pone.0030790 22292041 PMC3264639

[36] Brand Y, Levano S, Radojevic V, Naldi AM, Setz C, Ryan AF, Pak K, Hemmings BA, Bodmer D. All Akt isoforms (Akt1, Akt2, Akt3) are involved in normal hearing, but only Akt2 and Akt3 are involved in auditory hair cell survival in the mammalian inner ear. PLoS One. 2015; 10:e0121599. 10.1371/journal.pone.0121599 25811375 PMC4374771

[37] Pauley S, Lai E, Fritzsch B. Foxg1 is required for morphogenesis and histogenesis of the mammalian inner ear. Dev Dyn. 2006; 235:2470–82. 10.1002/dvdy.20839 16691564 PMC3901532

[38] Ding Y, Meng W, Kong W, He Z, Chai R. The Role of FoxG1 in the Inner Ear. Front Cell Dev Biol. 2020; 8:614954. 10.3389/fcell.2020.614954 33344461 PMC7744801

[39] Obendorf M, Meyer R, Henning K, Mitev YA, Schröder J, Patchev VK, Wolf SS. FoxG1, a member of the forkhead family, is a corepressor of the androgen receptor. J Steroid Biochem Mol Biol. 2007; 104:195–207. 10.1016/j.jsbmb.2007.03.012 17482455

[40] He ZH, Zou SY, Li M, Liao FL, Wu X, Sun HY, Zhao XY, Hu YJ, Li D, Xu XX, Chen S, Sun Y, Chai RJ, Kong WJ. The nuclear transcription factor FoxG1 affects the sensitivity of mimetic aging hair cells to inflammation by regulating autophagy pathways. Redox Biol. 2020; 28:101364. 10.1016/j.redox.2019.101364 31731101 PMC6920089

[41] He ZH, Li M, Fang QJ, Liao FL, Zou SY, Wu X, Sun HY, Zhao XY, Hu YJ, Xu XX, Chen S, Sun Y, Chai RJ, Kong WJ. FOXG1 promotes aging inner ear hair cell survival through activation of the autophagy pathway. Autophagy. 2021; 17:4341–62. 10.1080/15548627.2021.1916194 34006186 PMC8726647

